# Activity-based detection of synthetic cannabinoid receptor agonists in plant materials

**DOI:** 10.1186/s12954-024-01044-4

**Published:** 2024-07-01

**Authors:** Axelle Timmerman, Margot Balcaen, Vera Coopman, Maarten Degreef, Eline Pottie, Christophe P. Stove

**Affiliations:** 1 Laboratory of Toxicology, Department of Bioanalysis, Faculty of Pharmaceutical Sciences, Ghent University, Ghent, Belgium; 2 Belgian Early Warning System on Drugs, Unit Illicit drugs, Health information, Sciensano, Brussels, Belgium; 3 Eurofins Forensics Belgium, Bruges, Belgium

**Keywords:** Untargeted screening, CB_1_ cannabinoid receptor bioassay, Adulterated cannabis, Synthetic cannabinoid receptor agonists (SCRAs), New psychoactive substances (NPS), Harm reduction

## Abstract

**Background:**

Since late 2019, fortification of ‘regular’ cannabis plant material with synthetic cannabinoid receptor agonists (SCRAs) has become a notable phenomenon on the drug market. As many SCRAs pose a higher health risk than genuine cannabis, recognizing SCRA-adulterated cannabis is important from a harm reduction perspective. However, this is not always an easy task as adulterated cannabis may only be distinguished from genuine cannabis by dedicated, often expensive and time-consuming analytical techniques. In addition, the dynamic nature of the SCRA market renders identification of fortified samples a challenging task. Therefore, we established and applied an in vitro cannabinoid receptor 1 (CB_1_) activity-based procedure to screen plant material for the presence of SCRAs.

**Methods:**

The assay principle relies on the functional complementation of a split-nanoluciferase following recruitment of β-arrestin 2 to activated CB_1_. A straightforward sample preparation, encompassing methanolic extraction and dilution, was optimized for plant matrices, including cannabis, spiked with 5 µg/mg of the SCRA CP55,940.

**Results:**

The bioassay successfully detected all samples of a set (*n* = 24) of analytically confirmed authentic Spice products, additionally providing relevant information on the ‘strength’ of a preparation and whether different samples may have originated from separate batches or possibly the same production batch. Finally, the methodology was applied to assess the occurrence of SCRA adulteration in a large set (*n* = 252) of herbal materials collected at an international dance festival. This did not reveal any positives, i.e. there were no samples that yielded a relevant CB_1_ activation.

**Conclusion:**

In summary, we established SCRA screening of herbal materials as a new application for the activity-based CB_1_ bioassay. The simplicity of the sample preparation, the rapid results and the universal character of the bioassay render it an effective and future-proof tool for evaluating herbal materials for the presence of SCRAs, which is relevant in the context of harm reduction.

## Introduction

Synthetic cannabinoid receptor agonists (SCRAs) comprise a substantial portion of the new psychoactive substances (NPS) monitored by the EMCDDA (European Monitoring Centre for Drugs and Drug Addiction). NPS are substances designed to replicate the experiences induced by conventional recreational drugs, while possessing altered chemical structures to evade legislation and/or detection. Specifically for SCRAs, the psychoactive effects of Δ^9^-tetrahydrocannabinol (THC), the main psychoactive constituent of *Cannabis sativa*, are mimicked [1]. SCRAs were initially synthesized to explore the endogenous cannabinergic pathways and to serve as pharmaceutical agents for conditions such as pain, anorexia, glaucoma, muscle spasms and wasting syndrome. Their main mechanism of action relies on their interaction with the cannabinoid receptor 1 and 2 (CB_1_ and CB_2_) [2]. The activation of CB_1_, mostly present in the central nervous system, attributes to their pharmacological similarity to THC. Besides producing the desired THC-like effects, such as euphoria and relaxation, the potential ‘legality’ of these substance makes them an interesting alternative for cannabis in countries where its usage is prohibited [2–4].

In comparison to THC, SCRAs often have a higher potency and efficacy at CB_1_ [5], and may cause serious adverse effects, including agitation [6], cardiovascular problems [7], psychological disorders [8, 9], nausea and vomiting, depressed breathing, muscle twitches, acute renal failure, suicidal ideation and cognitive impairment [10, 11]. The relevance and associated risks have led to the classification of many SCRAs as controlled substances, with the legal status varying by country. They may be nominally listed in controlled substance schedules or may be regulated via generic structure classification, covering substances with a similar chemical framework, aiming at a more future-proof legislation [12, 13]. Because of these regulatory efforts, clandestine and other labs continue to introduce (subtle) structural alterations, resulting in a rapid turnover of the NPS market. The resulting broad structural variety has rendered routine structure-based immunological drug screens ineffective, as these lack adequate cross-reactivity and sensitivity in detecting these compounds in biological matrices or confiscated materials [2, 14]. Rapid, effective and future-proof screening methods are required to address this challenge. In particular, ‘untargeted’ screening strategies are considered interesting, as these do not necessarily rely on certified reference materials or continuously updated mass spectral or other libraries. High resolution mass spectrometry (HRMS) serves as the gold standard for NPS detection [15]. However, this analytical method is costly and time-consuming, limiting its routine implementation for high-throughput screening and in resource-limited settings [14, 16–18].

SCRAs are available in several forms, including as herbal preparations or laced tobacco for smoking purposes [3]. These preparations are typically the result of spraying or soaking plant material with SCRAs, dissolved in an organic solvent, followed by drying and crushing [2, 19–21]. Since 2004, Europe has witnessed the emergence of these preparations, often referred to as ‘Spice’ or ‘legal highs’, which are sold online or via specialized shops in colourful packages [12].

Low-THC cannabis is defined by the EMCDDA as “a product being or containing cannabis herb, resin, extracts or oils that claim or appear to have a very low percentage of THC and which would be unlikely to cause intoxication” [22]. Since 2019, and initially observed in Switzerland, a new SCRA-related phenomenon has emerged, in which low-THC cannabis is adulterated with SCRAs [23–25]. The rationale behind the adulteration of industrial (or low-THC) hemp seems evident from a manufacturer’s standpoint, given the lower cost associated with low-THC hemp cultivation and the visually similar appearance. Because of this similar appearance, people who use traditional cannabis may believe that they are buying and consuming authentic cannabis. This combination of the consumer’s unwitting use of SCRAs, along with the SCRAs’ heightened potency and toxicity compared to THC, increases the risk of (severe) intoxications and unanticipated health effects [2, 3, 5, 26, 27]. The need for a better understanding of this phenomenon was also highlighted by the EMCDDA, which expressed concerns regarding public health and consumer protection. In this context, from a harm reduction perspective it is relevant to be able to distinguish between ‘genuine’ cannabis and SCRA-adulterated cannabis, given the increased health risks posed by the latter.

Starting from a previously described in vitro activity-based CB_1_ assay, this study reports on a novel application of this assay to screen for cannabinoid activity in herbal products. The ‘untargeted’ activity-based methodology utilizes a CB_1_-β-arrestin 2 (βarr2) recruitment assay based on functional complementation of a split-nanoluciferase (NanoBiT®; NanoLuc® Binary Technology), facilitating real-time monitoring of protein interactions in living cells [28, 29]. Although there are currently several assays for measuring CB_1_ activation, the CB_1_-βarr2 recruitment assay offers several advantages such as its simplicity, real-time monitoring, and the absence of radioactivity. Moreover, it has proven to be a sensitive and selective system, with the measurement of a receptor-proximal event as a read-out (βarr2 recruitment to activated CB_1_), minimizing false positive scoring [30, 31]. The methodology was optimized using a diverse set of CP55,940-spiked plant matrices and suitability was demonstrated using a set of analytically confirmed authentic Spice products. The methodology was subsequently applied to screen an extensive set of cannabis/herbal products (*n* = 252), collected during the summer of 2022 at an international dance festival, to evaluate the prevalence of SCRA-laced products/adulterated cannabis.

## Materials and methods

### Chemicals and reagents

Seized ‘Spice-like’ herbal incenses were provided by Eurofins Forensics Belgium (Bruges, Belgium), previously analyzed as part of their forensic toxicology activities. The seized plant material from a Belgian international dance festival had been pooled and given for further investigation to the Belgian Early Warning System on Drugs. The reference standard of CP55,940 was purchased from Sigma-Aldrich (Darmstadt, Germany). Dulbecco’s modified Eagle’s medium (DMEM, supplemented with GlutaMAX™), OptiMEM® I Reduced Serum Medium, Penicillin-Streptomycin (10,000 IU/mL and 10,000 µg/mL) and amphotericin B (250 µg/mL) were purchased from Thermo Fisher Scientific (Waltham, Massachusetts, USA). Fetal bovine serum (FBS) and poly-D-lysine hydrobromide were supplied by Sigma-Aldrich. Methanol (MeOH) was purchased from Chem-Lab Analytical (Zedelgem, Belgium) and acetonitrile (ACN) was from Biosolve (Valkenswaard, The Netherlands). Nano-Glo® Live Cell reagent and the corresponding Nano-Glo LCS Dilution buffer were from Promega (Madison, Wisconsin, USA).

### Sample preparation

To obtain an extract compatible with the employed bioassay, the herbal materials potentially containing SCRAs underwent a simple and quick sample preparation. MeOH (1 mL) was added to approximately 20 mg of herbal material in 2 mL microtubes. The mixtures were vortexed for 2–5 s and sonicated for 5 min to extract potentially present SCRAs. After a 10 min centrifugation at 20,800 x g, 10 µL of the supernatant was transferred to a 1.5 mL microtube and diluted 1:100 in MeOH. This extract was further diluted 1:1 in OptiMEM®. All extracts and controls were freshly prepared on the day of the experiment.

### Cell culture and in vitro CB_1_ β-arrestin 2 recruitment assay

**Fig. 1:**
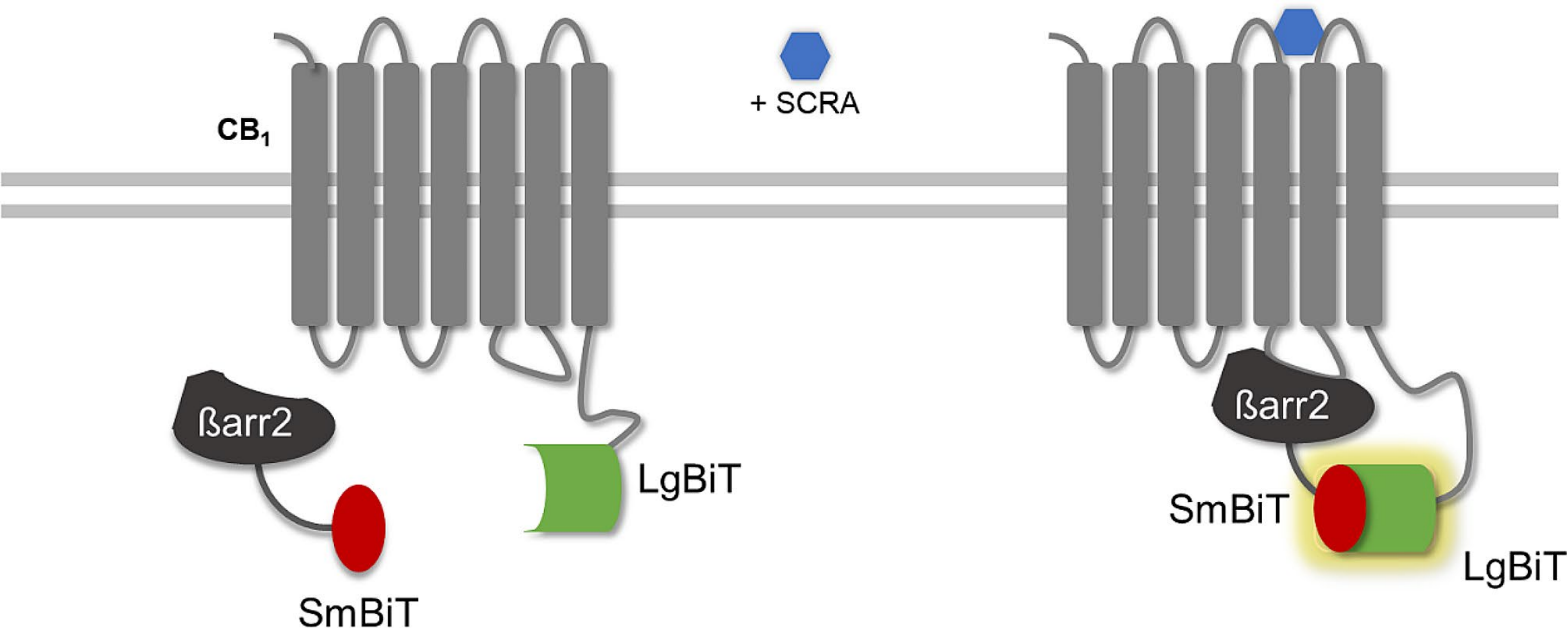
Set-up of the cell-based CB_1_ β-arrestin 2 (βarr2) recruitment assay. The presence of (a) SCRA(s) leads to activation of CB_1_, (fused to LgBiT), which initializes the recruitment of βarr2 (fused to SmBiT). Upon reassociation of the two subunits, a functional NanoLuc is restored, yielding a strong bioluminescent signal in the presence of the substrate, furimazine

Screening for the presence of SCRAs in herbal matrices was achieved with a live cell-based βarr2 recruitment assay to monitor CB_1_ activation. The generation of a cell line stably expressing CB_1_ and a truncated βarr2 (βarr2_TR366_) in the NanoBiT® system has been previously described [28–30, 32]. The assay principle relies on the functional complementation of a split-nanoluciferase following the recruitment of SmBiT-βarr2 to activated CB_1_-LgBit (SmBit and LgBit representing a small (1.3 kDa) and large (18 kDa) subunit of the split-nanoluciferase), as depicted in Fig. 1. A bright bioluminescent signal is subsequently generated in the presence of the enzyme’s substrate furimazine. Stable cell lines were used to reduce the workload and enhance the reproducibility within a set of experiments, while the truncated form of βarr2 improves the analytical sensitivity of the bioassay [29, 30].

Human embryonic kidney (HEK) 293T cells, stably expressing the above-mentioned constructs, were routinely cultured at 37 °C and 5% CO_2_ in a humidified environment. Cells were grown in DMEM (GlutaMAX™), supplemented with 10% heat-inactivated FBS, 100 mg/L streptomycin, 0.25 mg/L amphotericin B and 100,000 IU/L penicillin. For the experiments, a two-day protocol was followed, in which the cells were seeded (5 × 10^4^) in white, poly-D-lysine coated 96-well plates on the first day. Following overnight incubation, cells were rinsed twice with 150 µL OptiMEM® to remove remaining medium, after which 100 µL OptiMEM® was added to each well. Twenty-five µL of the diluted Nano-Glo® Live Cell Reagent substrate (prepared following the manufacturer’s protocol, i.e., 20-times diluted in the Nano-Glo® LCS Dilution Buffer) was then pipetted into each well. Subsequently, the plate was placed in the TriStar² LB 942 Multimode Microplate Reader (Berthold Technologies GmbH & Co., Germany), to monitor luminescence until stabilization of the signal (4–5 cycles). Next, 10 µL of the 200-fold diluted extract solution was pipetted into each well, followed by a 2 h (real-time) measurement (although scoring was already possible after 30 min). Appropriate controls were included in all experiments. The final 3.7% MeOH in-well concentration doesn’t interfere with the assay because of the short readout-time of the assay [28].

### Liquid chromatography coupled to time-of-flight mass spectrometry (LC-QTOF-MS)

‘Spice-like’ herbal incenses, initially analyzed by gas chromatography mass spectrometry, were reanalyzed by LC-QTOF-MS. Chromatographic separation was performed with an Agilent 1290 Infinity LC system coupled to a Phenomenex Kinetex C18 column (2.6 μm; 3 mm x 50 mm), maintained at 30 °C. The high resolution mass spectrometry system (HRMS) was a 5600 + QTOF from Sciex (Framingham, MA, USA), with an electrospray ionization (ESI) source and using Sciex Analyst TF 1.8.1 software to manage the system. The LC-HRMS settings were the same as those previously published [33, 34], except for the start and duration of the LC gradient (starting at 50% solvent B, with a linear increase to 98% solvent B in 5 min) and the mass ranges (scanning from 250 to 500 Da for the TOF-MS full scan spectra, combined with data-dependent acquisition of product ion spectra (scanning from 50 to 500 Da)). A 10 µL aliquot of a 1:1000 dilution of the initial methanolic extract in diluent (12.5% 50/50 ACN/MeOH in water) was injected. The respective peak intensities of the identified SCRAs present in a given extract were used to obtain a rough estimation of the relative contribution of each SCRA within a Spice product. For this purpose, I_SCRA_/ I_total_ was calculated as the ratio of the peak intensity for a given SCRA (I_SCRA_) to the overall peak intensities of all the SCRAs detected in a preparation (I_total_), using the HighResNPS library with a cutoff set at 50.

### Data analysis

Microsoft Excel 2019 was used to plot time-luminescence profiles and analyze the data, as described before [30]. To account for inter-well variability, absolute signals from the last equilibration cycle were used for correction of the raw luminescence values. Subsequently, all data points were baseline corrected by subtraction of the corresponding average of the solvent controls. Average corrected time-luminescence values with error bars (standard error of mean, SEM) for three independent experiments or duplicates for one representative experiment were plotted using GraphPad Prism Software (Version 10.0.0). The results obtained in the bioassay were scored independently by two individuals, based on the obtained sample profiles after correction with the signal obtained for the solvent control. Samples were only scored positive when the relative light unit (RLU) signal intensities exceeded 50% of the positive control. These RLU intensities are correlated with the level of CB_1_ activation, which depends on the activity of the SCRA (i.e. potency and efficacy) and the dose of the SCRA used to make the preparation.

## Results

### Optimization of the methodology for screening of herbal products

Screening of plant material for the presence of SCRAs using the CB_1_ activity-based assay requires the samples to be in the format of an extract. The wide variety of herbal matrices that can potentially be used as carrier material for the deposition of SCRAs requires a robust procedure. In other words, the sample preparation must be applicable to all herbal matrices. In essence, we aimed at finding a universally applicable sample preparation procedure that met the following three criteria: (i) compatibility with the CB_1_ bioassay, (ii) simplicity, and (iii) uniformity of the procedure for diverse herbal matrices. Importantly, the sample preparation aims at extracting SCRAs *on* the plant material, rather than extracting (possibly present THC) *from* the material (e.g. the material is not homogenized in a mortar). In pursuit of this aim, optimization experiments were conducted using a diverse set of plant matrices, encompassing pure cannabis, mixtures of cannabis with tobacco, or undefined dry plant material (*n* = 7).

Twenty mg of each matrix was spiked with 200 µL of a 0.5 mg/mL solution of CP55,940 (~ 5 µg/mg, ~ 0.5% w/w), a SCRA commonly used as a reference in CB_1_ activity-determination studies [35–37]. In the assay used here, CP55,940 only shows a moderate efficacy at CB_1_ and a spiking level of 0.5% was chosen to yield a signal that was sufficiently distinguishable from background, yet was not too high (most currently encountered SCRAs have an efficacy that is much higher than that of CP55,940) [35, 36, 38–40]. Importantly, the chosen level of spiking can be considered relatively low for Spice, as a typical content found in Spice products varies from 1.67 to 10% w/w [41], and the level is thereby within the range of spiking observed in adulterated low-THC, with levels from 0.2 to 0.56% w/w [26]. Initially, a 1:1 dilution of methanolic extract in OptiMEM® was directly applied in the assay. Notably, while all methanolic extracts were transparent (though often colored, i.e. yellowish or greenish), the 1:1 dilution in OptiMEM® often turned into a turbid liquid that considerably interfered with the assay read-out. This interference could be mitigated by a 1:100 dilution of the extract with MeOH, followed by a 1:1 dilution in OptiMEM®.

**Fig. 2:**
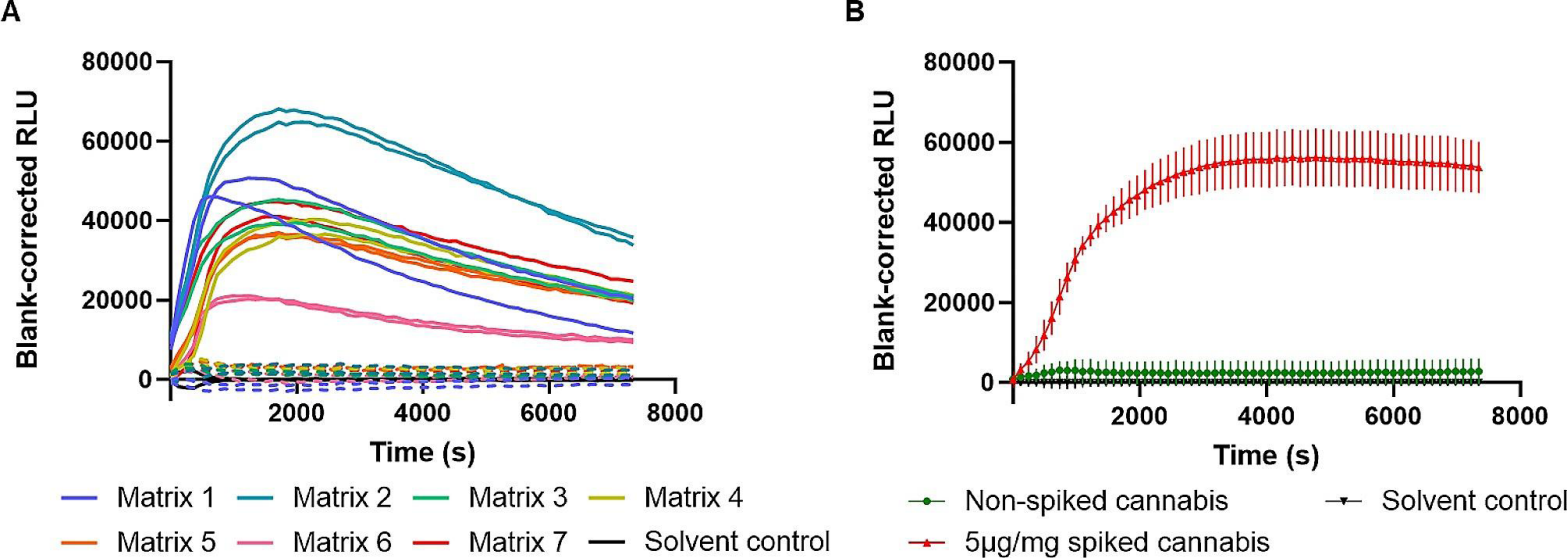
(**A**) CB_1_ activation profiles of a diverse set of non-spiked (dashed lines) and spiked (5 µg CP55,940/mg) plant matrices (*n* = 7) (full lines). Data are presented as duplicates from one representative experiment. (**B**) CB_1_ activation profiles of non-spiked (green) and spiked (5 µg CP55,940/mg) cannabis (red). The activation profiles represent the average blank-corrected luminescence ± standard error of the mean (SEM) of three independent experiments (*n* = 3), each performed in duplicate. Solvent control (50:50 OptiMEM®/MeOH; black) was included in each panel

All seven CP55,940-spiked plant matrices yielded CB_1_ activation profiles that exhibited a distinct increase in RLU when compared to their corresponding non-spiked matrix and blank (OptiMEM®/MeOH (50:50, v/v)) (Fig. 2A). This indicates that the optimized sample preparation was generally suited to extract this SCRA from different plant matrices. One of the CP55,940-spiked plant matrices (matrix 7, corresponding to an authentic cannabis sample) was included as a positive control in the following experiments. Given the fact that we deliberately did not aim at (an efficient) extraction of THC *from* cannabis, we cannot exclude that THC and other phytocannabinoids (like Δ^9^-tetrahydrocannabinolic acid (THCA) and cannabidiol (CBD)) may be co-extracted to some extent. However, previous studies have shown that CBD does not result in CB_1_ activation, and THCA requires decarboxylation to yield the active THC [28, 42, 43]. With the assay set-up used here, THC itself, which may also readily be present in cannabis material, is only a weak partial agonist at CB_1_, with an efficacy < 10% compared to that of CP55,940, while CP55,940 itself is also only a moderately efficacious agonist relative to other SCRAs [44]. In combination with the threshold used here, arbitrarily set at 50% of the 0.5% w/w-spiked CP55,940 control, even high-THC cannabis is anticipated to result in a negative scoring. This is also in line with the fact that non-spiked cannabis resulted in a negative scoring in the bioassay (Fig. 2B) [44].

### Activity-based screening of authentic spice-like herbal incenses in the CB_1_ bioassay

A total of 24 confiscated authentic Spice materials were used to evaluate the applicability of the bioassay. Twenty-one of these represented different Spice products, i.e. both the herbal material and the SCRA(s) they contained differed. According to seizure data, the remaining three samples originated from a single batch production, i.e. the herbal material and SCRA(s) were expected to be the same for these samples. The materials were present in colorful, airtight metal-foil sachets, printed with typical labels such as “not for human consumption”, “adults only” and “use responsibly”. Although the packaging provided details about the herbal content (e.g. *Tribulus Terrestris*, *Stevia* leaf, *Leonorus Sibricus*, …), information regarding SCRAs was lacking. This created the impression that the product was legal, as the labelling suggested it originated solely from natural sources.

LC-HRMS was performed for SCRA identification. The HighRes NPS library [45] and a set of in-house reference standards were used to include matching based on (i) mass accuracy, (ii) retention time, (iii) isotope pattern, and (iv) MS² spectrum (number of fragment ions) in the identification procedure. Based on the analytical findings, the majority of Spice products were found to contain blends of various SCRAs. More specifically, the following SCRAs were most commonly identified: JWH-210, JWH-122, JWH-018, AM-2201, 5 F-MDMB-PICA (5 F-MDMB-2201), and AMB-FUBINACA (FUB-AMB) or a structural isomer. Table 1 provides a rough estimation of the relative contribution of each SCRA within a Spice product, based on their respective peak intensities. While an important caveat of this procedure is that a direct comparison of the relative abundance of SCRAs within a given product is challenging due to the varying ionization efficiencies of each SCRA, this approach did allow to distinguish between major and minor contributors in a given product.

**Fig. 3:**
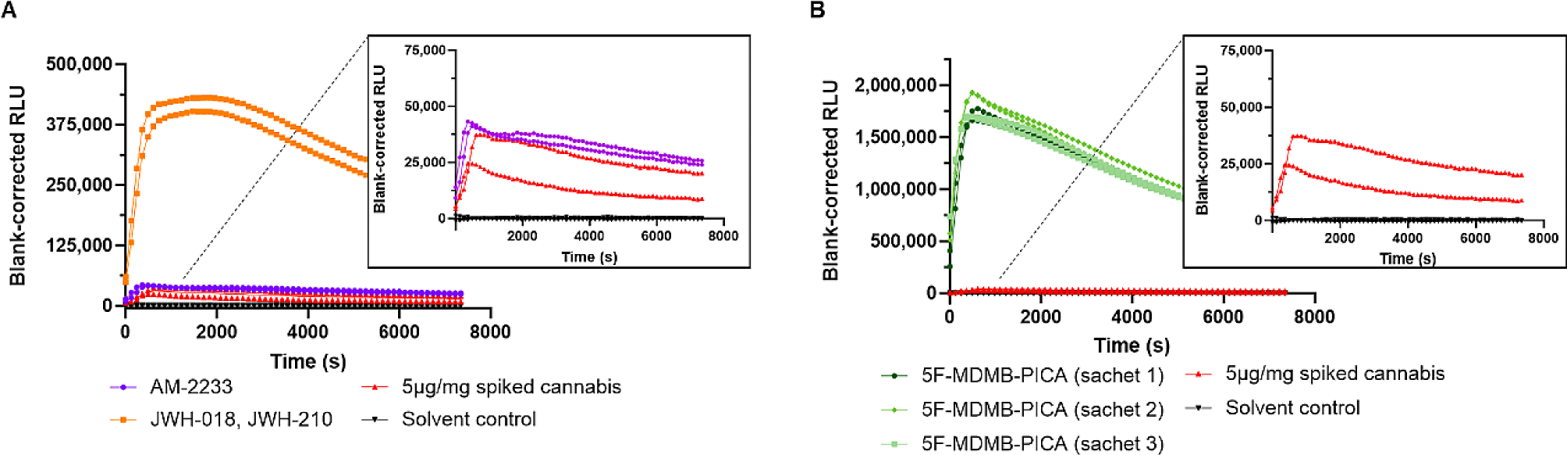
CB_1_ activation profiles obtained for extracts from authentic Spice products. (**A**) Comparison of two Spice products, with a zoom-in of the activation profile induced by AM-2233-containing Spice. (**B**) Comparison of different sachets containing 5 F-MDMB-PICA Spice, originating from the same production batch. The positive control (5 µg/mg CP55,940-spiked cannabis (red)) is indicated in every panel. All profiles originate from the same experiment, with duplicates per condition. Note the differences in scaling

Extracts from all 24 Spice products resulted in CB_1_ activation, as evident from a clear but widely varying increase in RLU, confirming the effectiveness of the utilized approach. Figure 3A depicts the activity data of an AM-2233-containing Spice sample (purple traces) yielding a signal similar to 5 µg/mg CP55,940-spiked cannabis. Conversely, a much higher signal was obtained for a Spice product containing JWH-018 and, to a much lesser extent, JWH-210 (orange traces, estimated relative abundances are shown in Table 1).

Figure 3B depicts the activation profiles induced by extracts from three 5 F-MDMB-PICA-containing Spice products stemming from the same ‘production batch’. The overlapping activation profiles readily suggest that these samples may have a similar composition. This was corroborated by the analytical data, showing similar peak intensities for 5 F-MDMB-PICA (Table 1). The fact that for these products the extent of CB_1_ activation greatly surpassed the signal from the 5 µg/mg CP55,940-spiked cannabis, strongly indicates the strength (in terms of CB_1_ activation potential) of these Spice products. Moreover, the consistent (analytical and bioassay) data also support the robustness of the extraction.

**Table 1:** Analytical results for the identified SCRA(s) in selected Spice products

Figure	Spice sample	Identified SCRA	Peak intensity identified SCRA (I_SCRA_)	Total peak intensity (I_total_)	Relative percentage*
**3 A**	1	AM-2233	6,145,342	6,335,616	97.0%
	2	JWH-018 JWH-210	17,099,541 832,163	18,297,257	93.5% 4.6%
**3B**	3	5 F-MDMB-PICA	11,038,011	11,923,126	92.6%
	4	5 F-MDMB-PICA	12,224,551	13,219,990	92.5%
	5	5 F-MDMB-PICA	10,645,021	11,438,299	93.1%

*The estimated relative percentage was calculated using the formula: I_SCRA_/ I_total_

### Activity-based screening of a large set of seized herbal materials from an international dance festival

**Fig. 4:**
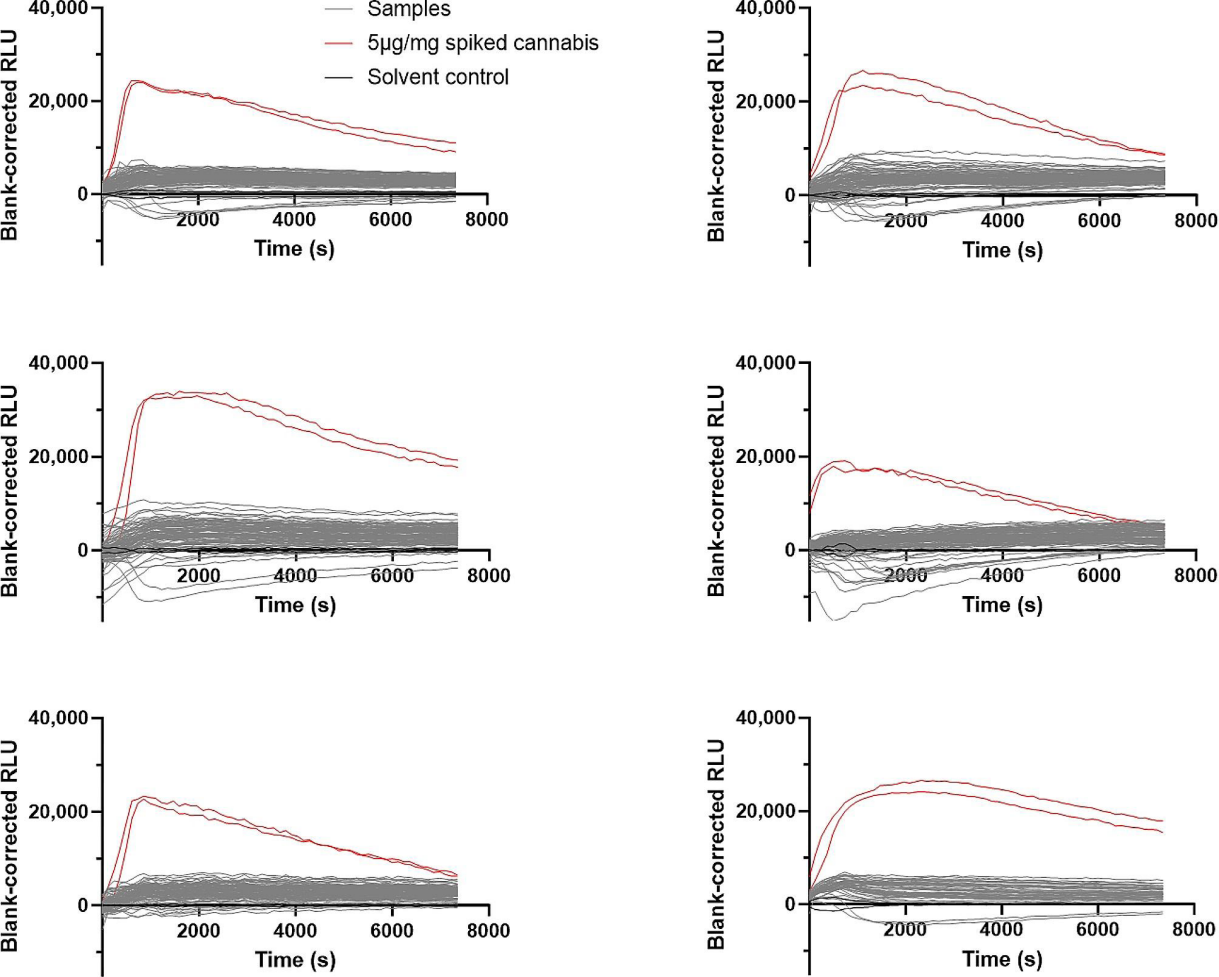
Outcome of the CB_1_ analyses performed on a large set (*n* = 252) of confiscated herbal materials. Samples (grey) were compared to solvent control (black) and CP55,940-spiked cannabis as positive control (red). Data are presented as duplicates from six experiments, with 46 samples per plate

Having confirmed the applicability of the bioassay for the screening of herbal products, we subsequently applied the methodology on a large collection (*n* = 252) of confiscated herbal products, comprising a diverse range of plant matrices, such as tobacco, dried herbs mixed with cannabis or solely cannabis. All extracts were measured in duplicate, and each experiment included a positive control and a solvent control, as specified earlier. Although some activation profiles deviated slightly from the solvent control (with both minor increases or decreases in RLU being observed), these fluctuations were likely solely related to the complex nature of these samples. Importantly, the profile of none of the samples approached that of the positive control (which, as specified earlier, readily represents a low-level of spiking with only a moderately efficacious SCRA). Hence, none of the confiscated herbal materials was scored positive in terms of SCRA spiking (Fig. 4). In other words, we could not conclude the presence of SCRAs in any of these samples. Nevertheless, it cannot be excluded that plant materials, spiked with a low dose of (a) low efficacy SCRA(s), might be scored false negative.

## Discussion

At present, common ‘targeted’ and ‘untargeted’ analytical techniques encounter challenges when attempting to detect SCRAs in biofluids and plant-based matrices. This difficulty mainly arises from the continuous structural modifications of these substances, aiming at evading detection and legal regulation [1, 30]. In previous research, we reported on the development and application of an activity-based screening principle, capable of monitoring the CB_1_ activating potential of extracts from biological samples [29, 30]. So far, this method has been applied for the characterization of reference materials and for the screening of biological matrices and powders for the presence of SCRAs, irrespective of the chemical structure of these SCRAs [35–37]. This study aimed at evaluating the potential of our bioassay to assess the relevant presence of SCRAs in herbal materials in a simple and universal manner, as from a harm reduction perspective it is relevant to be able to distinguish ‘genuine’ from adulterated cannabis.

To produce an extract compatible with the CB_1_ bioassay format, a straightforward extraction-and-dilution sample preparation procedure, not requiring any sophisticated lab equipment, was set up. Additionally, a user-friendly luminometer was utilized for the read-out, offering a notable contrast to the often highly complex analytical instruments and their data analysis. Inclusion of a dilution step was required to cope with matrix effects that may obscure the detection of SCRAs and can be linked to the complexity of herbal matrices, such as the commonly used Lamiaceae Mint herbs (*Mentha*, *Mellissa* and *Thymus*) and *Turnera diffusa* [1, 46–48].

Suitability of the proposed sample preparation was evaluated by assessing herbal materials spiked with CP55,940, typically used as a reference SCRA in SCRA pharmacology research. In our activity-based assays, CP55,940 shows a potency in the sub-nanomolar range (EC_50_ = 0.48 nM) but an efficacy that is generally lower than that observed with members of other SCRA classes, like JWH analogues [35, 38]. Theoretically, also other (more efficacious) SCRAs could be used as a reference, pending spiking at an appropriate level, to yield a signal clearly distinguishable from solvent control, while not being too high. Here, a spiking level of 0.5% w/w of CP55,940 in herbal material was chosen to optimize sample preparation, which is below spiking levels typically seen in conventional Spice products (i.e. 1.67-10% w/w) [41, 49–51]. However, in adulterated low-THC cannabis also lower SCRA/plant ratios have been observed [26]. Lowering the dosage of SCRAs in adulterated cannabis products, as compared with the typical dosing seen in Spice preparations, might be explained by the intended purpose of these adulterated (low-THC) hemp products, i.e. mimicking the effects of genuine cannabis [26].

Our data demonstrated that, by incorporating a 100-fold dilution, the CB_1_ activity-based assay easily detected a 0.5% w/w spiking of CP55,940 in each of the seven distinct plant materials. In the absence of spiking, none of the plant materials, including genuine cannabis, exhibited clear CB_1_ activation. The absence of a relevant signal in unadulterated cannabis extracts was both desired and expected and is related to the fact that the extraction procedure does not aim at extracting THC, and that THCA, the THC precursor present in plant material, requires exposure to heat (e.g. during smoking) to undergo decarboxylation to form the partial CB_1_ agonist THC [42, 43, 52].

Next, the method’s effectiveness was evaluated using a panel of authentic Spice products that contained either a single SCRA or a blend of multiple SCRAs. Extracts from all Spice products showed outspoken CB_1_ receptor activation, resulting in a positive scoring. In addition to demonstrating its potential to serve as a screening tool, the bioassay offered insights into (i) the ‘strength’ of a Spice product, as indicated by its CB_1_ activation potential, serving as an indicator of its potential harm, and (ii) whether various samples may have originated from different batches or possibly the same batch.

The bioassay was able to distinguish different strengths in Spice compositions, based on distinct CB_1_ activation profiles, the latter being determined by the identity and concentration of the SCRA being present. As an illustration, an AM-2233-spiked Spice product yielded a signal comparable to that exerted by our control (~ 0.5% w/w) CP55,940-spiked cannabis, indicating a similar strength of these preparations. Other products yielded much higher signals. E.g., the much stronger CB_1_ activation potential of JWH-018/JWH-210 containing Spice suggests that this is a stronger Spice preparation, which aligned well with the obtained analytical data (with a lower peak intensity being observed for AM-2233), and with the fact that a higher intrinsic CB_1_ activation potential has been observed for both JWH SCRAs than for AM-2233 [28, 35, 53, 54]. While it can reasonably be assumed that ‘consumption’ of products with a higher strength will result in more pronounced CB_1_-related effects in vivo, the RLU cannot be directly translated to in vivo toxicity.

According to studies by Moosmann et al. [51] and Shanks et al. [55], manufacturers easily change SCRA formulations in ‘legal high’ products to bypass legislation while maintaining the same brand/packaging. This switch may involve selecting a SCRA with a potentially higher CB_1_ activating potential, while maintaining the ratio SCRA/plant material, which may lead to unanticipated effects and harms for the people who use these drugs. The bioassay may hold significant value in this context as it can assess the extent to which a Spice product (also when it contains a mix of SCRAs) can activate CB_1_, allowing for an estimation of the potential harm associated with the product, even without identifying the SCRA(s) present. In fact, one could envisage that in the future a standardized product, laced with a particular SCRA (CP55,940, JWH-018, or another SCRA) could serve as a reference, against which the activity in other products could be compared. This shows resemblance to the principle of ‘activity equivalents’, as proposed for assisting in the interpretation of SCRA or opioid preparations and intoxications [56–58].

In addition to the evaluation of the collective CB_1_ activity of herbal products, the bioassay also demonstrated its utility in identifying samples that might originate from the same production batch. In the samples coming from a single batch that were evaluated here, the overlapping activation profiles (Fig. 3B) readily suggested a potentially similar composition, which was corroborated by the analytical data. While, obviously, similar profiles may coincidentally also be obtained from different batches, containing different SCRAs with different potencies or efficacies, different profiles readily allow to discern distinct batches.

Lastly, the CB_1_ bioassay was employed to screen a set of 252 herbal samples, primarily cannabis, gathered at an international dance festival during the summer of 2022. This screening endeavor aimed at determining whether the phenomenon of adulterating herbal material (mainly cannabis) with SCRAs would be prevalent in Belgium. This is relevant to gain insight into the concept of potential ‘unwitting consumption’ in a harm reduction context. Interestingly, application of the bioassay did not yield any positive results (i.e. no sample reached the imposed threshold of 50% of the RLU from the positive control), indicating that none of the evaluated materials was spiked with SCRAs (at least, not to a relevant extent). While we cannot fully exclude that low levels of activity may have been present in these samples, this would be less relevant from a harm reduction perspective, as authentic cannabis preparations -when smoked- will also result in cannabinoid effects. Overall, considering the limited in vitro activation potential of the tested samples, we can cautiously conclude that none of the 252 seized samples is expected to induce an overly strong CB_1_ activation.

In essence, the detection of SCRAs in diluted extracts from herbal material depends on the combination of relevant (sufficiently high) concentrations of SCRAs and the inherent CB_1_ receptor activation potential of those SCRAs. While this could be seen as a limitation, it is also a strength, as the assay does not aim to flag authentic (non-spiked) cannabis samples as ‘suspicious’ (i.e. spiked). Moreover, because of its activity-based nature, the methodology inherently detects current and potentially emerging SCRAs, establishing it as a future-proof screening method. As shown above, the currently employed bioassay effectively screens for the presence of relevant SCRA-positive samples and is capable of providing a result in less than 30 min. In practice, this methodology could even function as an on-site screening tool, as no highly sophisticated equipment (such as a mass spectrometer) is required. Moreover, the capacity of 46 samples each 30 min (with also sample preparation taking just a few minutes) allows a fast turnaround and is compatible with high-throughput screening of suspicious plant materials.

## Conclusion

To conclude, we extended here the application potential of activity-based CB_1_ bioassays by demonstrating its suitability to screen different herbal matrices for the presence of SCRAs. Following optimization of sample preparation using different CP55,940-spiked (0.5% w/w) plant matrices, the bioassay was demonstrated to be able to provide relevant information about the CB_1_ activation potential of SCRA(s) from Spice preparations. In addition, the CB_1_ activation profiles obtained in the bioassay for different samples could be used as a first criterion for tentative batch identification. Lastly, application on a large set (*n* = 252) of cannabis/plant materials sourced at an international dance festival in 2022 indicated the absence (at least to a relevant extent) of SCRAs. In summary, the simple sample preparation in combination with the high-throughput potential and the universal character of the bioassay, makes this methodology an effective, future-proof potential first-line screening tool that complements traditional analytical techniques.

## Data Availability

The datasets used and/or analysed during the current study are available from the corresponding author on reasonable request.

## Abbreviations

ACN: Acetonitrile
βarr2: β-arrestin 2
CB_1_: Cannabinoid receptor 1
CBD: Cannabidiol
DMEM: Dulbecco’s modified Eagle’s medium
EMCDDA: European Monitoring Centre for Drugs and Drug Addiction
ESI: Electrospray ionization source
FBS: Fetal bovine serum
HEK: Human embryonic kidney
HRMS: High resolution mass spectrometry
LC-HRMS: Liquid chromatography-high resolution mass spectrometry
LC-QTOF-MS: Liquid chromatography coupled to time-of-flight mass spectrometry
LgBiT: Large BiT, subunit of nanoLuc luciferase
MeOH: Methanol
NanoBiT®: NanoLuc^®^ Binary Technology
NanoLuc: Nanoluciferase
NPS: New psychoactive substances
RLU: Relative light unit
SCRA: Synthetic cannabinoid receptor agonist
SEM: Standard error of mean
SmBiT: Small BiT, subunit of nanoLuc luciferase
THC: Δ^9^-tetrahydrocannabinol
THCA: Δ^9^-tetrahydrocannabinolic acid
